# Quantitative assessment of simultaneous F-18 FDG PET/MRI in patients with various types of hepatic tumors: Correlation between glucose metabolism and apparent diffusion coefficient

**DOI:** 10.1371/journal.pone.0180184

**Published:** 2017-07-03

**Authors:** Eunjung Kong, Kyung Ah Chun, Ihn Ho Cho

**Affiliations:** Department of Nuclear Medicine, Yeungnam University Medical School and Hospital, Daegu, Republic of Korea; Kyungpook National University School of Medicine, REPUBLIC OF KOREA

## Abstract

**Purpose:**

Metabolism and water diffusion may have a relationship or an effect on each other in the same tumor. Knowledge of their relationship could expand the understanding of tumor biology and serve the field of oncologic imaging. This study aimed to evaluate the relationship between metabolism and water diffusivity in hepatic tumors using a simultaneous positron emission tomography/magnetic resonance imaging (PET/MRI) system with F-18 fluorodeoxyglucose (FDG) and to reveal the metabolic and diffusional characteristics of each type of hepatic tumor.

**Methods:**

Forty-one patients (mean age 63 ± 13 years, 31 male) with hepatic tumors (18 hepatocellular carcinoma [HCC], six cholangiocarcinoma [CCC], 10 metastatic tumors, one neuroendocrine malignancy, and six benign lesions) underwent FDG PET/MRI before treatment. Maximum standard uptake (SUVmax) values from FDG PET and the apparent diffusion coefficient (ADC) from the diffusion-weighted images were obtained for the tumor and their relationships were examined. We also investigated the difference in SUVmax and ADC for each type of tumor.

**Results:**

SUVmax showed a negative correlation with ADC (r = -0.404, p = 0.009). The median of SUVmax was 3.22 in HCC, 6.99 in CCC, 6.30 in metastatic tumors, and 1.82 in benign lesions. The median of ADC was 1.039 × 10^−3^ mm/s^2^ in HCC, 1.148 × 10^−3^ mm/s^2^ in CCC, 0.876 × 10^−3^ mm/s^2^ in metastatic tumors, and 1.323 × 10^−3^ mm/s^2^ in benign lesions. SUVmax was higher in metastatic tumors than in benign lesions (p = 0.023). Metastatic tumors had a lower ADC than CCC (p = 0.039) and benign lesions (p = 0.004). HCC had a lower ADC than benign lesions, with a suggestive trend (p = 0.06).

**Conclusion:**

Our results indicate that SUVmax is negatively correlated with ADC in hepatic tumors, and each group of tumors has different metabolic and water diffusivity characteristics. Evaluation of hepatic tumors by PET/MRI could be helpful in understanding tumor characteristics.

## Introduction

The liver is an important organ from the oncologic perspective. Primary hepatic tumors are particularly common in the presence of underlying chronic liver diseases [1]. Further, the liver is one of the most common organs for cancer metastasis. With the extensive use of anatomic imaging modalities, i.e., ultrasound and computed tomography (CT), there has been a considerable increase in the number of hepatic lesions identified incidentally. Further, up to 20% of individuals have a benign hepatic lesion, the most common being cavernous hemangioma and focal nodular hyperplasia [2].

F-18 fluorodeoxyglucose (FDG) positron emission tomography (PET)/ CT is used routinely to diagnose and stage disease and to evaluate the response to treatment for many cancers [3,4]. Cancer cells have increased intracellular accumulation of the glucose analog FDG due to increased glucose uptake and glycolysis. Glucose uptake in cancer cells is regulated by hypoxia, oncogenes, and growth factors. The maximum standardized uptake value (SUVmax) is used as a measure of FDG uptake; it is correlated with histologic grade [5] and other histopathologic features, such as mitotic count and presence of necrosis [6]. Diffusion-weighted imaging (DWI) in magnetic resonance imaging (MRI) is also increasingly used in tumor evaluation. Brownian motion of water molecules in tissue can be quantified by the apparent diffusion coefficient (ADC). Any structural changes in the proportion of extracellular to intracellular water protons or pathophysiologic state of the tissues will alter the ADC [7]. Like the SUV from PET, the ADC has been used clinically to differentiate benign from malignant tumors [8,9] and to assess tumor aggressiveness, characterization subtype, and predict prognosis [10–12].

Since both SUVmax and ADC provide information on tumor aggressiveness, some degree of correlation between these two quantitative imaging parameters could be expected. In this study, we assessed the relationship between the SUVmax and ADC in hepatic tumors using a simultaneous PET/MRI hybrid imaging system. This system can assess the relationship between SUVmax and ADC accurately by minimizing limitations such as patient motion and potential physiologic and treatment changes between separate PET and MRI examinations. Further, we investigated whether the quantitative parameters of FDG PET/MRI can be used to characterize hepatic tumor subtypes.

## Methods

### Subjects

Between May 2013and July 2015, 86 patients at our institution underwent abdominal PET/MR scan for assessment of a hepatic mass after detection on contrast-enhanced CT for staging or follow-up of a malignant disorder. We excluded patients who had received treatment before their scan or had multiple masses of a similar size and shape. A reference standard was established by biopsy, clinical follow-up, and/or additional imaging modalities. The study protocol was approved by the institutional review board at Yeungnam University Hospital in Daegu, Korea (YUH -13-0430-O57). And all patients gave written informed consent.

### Simultaneous PET/MR data acquisition

Patients fasted for at least 6 h beforehand and their blood glucose levels were required to be < 8.9 mmol/L before injection of FDG (381.1 ± 74 MBq). Abdominal PET/MR (Biograph mMR; Siemens Healthcare, Erlangen, Germany) following PET/CT acquisition was initiated at 90–120 minutes after tracer injection. Abdominal PET/MR was performed covering one bed position of liver using an approved surface coil for PET/MR without further FDG injection. The PET data acquisition was performed over 10 minutes and MR imaging was obtained at the same time using the following sequence protocol (Table 1). For quantitative analysis, ADC maps were automatically acquired by the MRI system using 3 b factors (50, 400 and 800 s/mm^2^). A three-dimensional ordered-subsets expectation maximization iterative reconstruction algorithm was applied with two iterations and 21 subsets for PET data. A 172×172 matrix was used.

**Table 1:** Technical parameters of MR sequences used in study.

	A coronal 3D VIBE	Axial and coronal HASTE	An axial SS- SE- EPI DWI
**TR**	3.6 ms	1000 ms	7700 ms
**TE**	TE1 1.23 ms, TE2 2.46 ms	67 ms	75 ms
**slice thickness**	3.12 mm	4 mm	6 mm
**matrix size**	172 × 172	180 × 320	83 × 128
**FOV**	500 mm	400 mm	380 mm
**GRAPPA acceleration factor**	2	2	2
**protocol time**	0:19	0:54	2:42
**others**	Dixon-based attenuation correction	free-breathing, delineation of anatomy and gross pathology	free-breathing, b-values 50, 400 and 800 s/mm^2^

3D VIBE, three-dimensional volume-interpolated breath-hold examination; HASTE, half-Fourier acquisition single-shot turbo spin-echo; SS, single-shot; SE, spin echo; EPI, echo planar imaging; DWI, diffusion weighted imaging; TR, repetition time; TE, echo time; FOV, field of view; GRAPPA, generalized auto-calibrating partially parallel acquisition

### Image analysis

**Fig 1:**
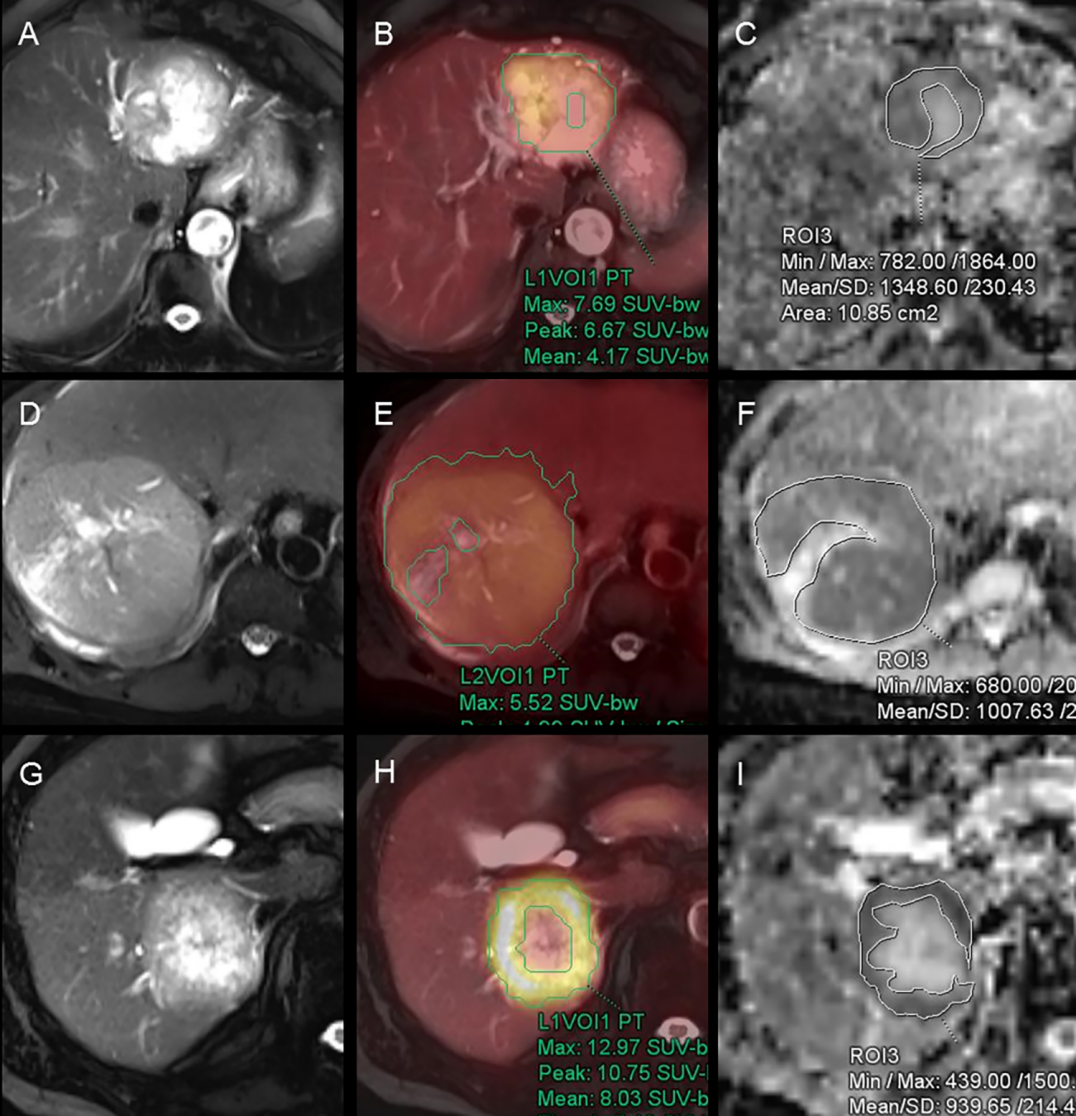
F-18 fluorodeoxyglucose (FDG) positron emission tomography (PET)/ magnetic resonance (MR) images with region of interest (ROI). (A–C) FDG PET/MR images of 85-year-old woman with cholangiocarcinoma; (D–F) 46-year-old man with hepatocellularcarcinoma; (G–I) 72-year-old man with hepatic metastasis from colon cancer. (A, D and G) Axial HASTE MRI images; (B, E and H) Fused images of FDG PET and HASTE; (C, F and I) ADC map. ROIs were manually drawn along the contour of the tumor except necrosis. HASTE, half-Fourier acquisition single-shot turbo spin-echo; ADC, apparent diffusion coefficient.

The PET and MR data sets were retrospectively analyzed by two nuclear medicine physicians on the PET/MR workstation (Syngo.via, Siemens Healthcare). In each case, a volumetric region of interest (ROI) was drawn over the entire area of uptake in the hepatic mass viewed on the axial fused PET/MR images. SUVmax values were calculated for each tumor and recorded automatically on the workstation (Fig 1).

The ROI for the ADC value was manually drawn to cover as much of the tumor as possible while avoiding necrosis, scars, and artifacts on the axial half-Fourier acquisition single-shot turbo spin-echo (HASTE) image along the largest cross-sectional diameter. The copying of the ROI from the HASTE into the corresponding DWI and ADC map enabled exact matching without the risk of incorrect positioning and erroneous workstation sampling. The ADC values were calculated as the average values of the pixels present in the ROI. Placement of the ROI was determined by consensus between two observers who were unaware of the clinical and histologic information pertaining to each case (Fig 1). This methodology limits intraobserver variability in ADC measurement, which was validated by performing the procedure on two separate occasions using randomly selected patients as a quality control measure. We analyzed the correlation between SUVmax and ADC and the difference between each of tumor subtype.

### Statistical analysis

Descriptive data are presented with mean ± standard deviation for continuous parametric variables, and median and range (min, max) for continuous nonparametric data. To evaluate the correlation between SUVmax and ADC, Pearson's and Spearman's rank correlation were performed. Pearson’s correlation was used for overall hepatic tumors and tumor subtypes with normally distributed variables such as hepatocellular carcinoma (HCC). Spearman's rank correlation was used for tumor subtypes with non-normally distributed variables such as metastatic tumors. To examine whether the SUVmax or ADC were significantly different between malignant and benign tumors, and among the tumor subtypes, Mann-Whitney test and Kruskal-Wallis tests were performed, respectively. Post hoc analyses were performed to further evaluate differences in the subtypes using Hodges-Lehmann estimator based on Mann-Whitney tests. The statistical analyses were performed using SPSS version 20 software (IBM Corp., Armonk, NY, USA). Statistical significance was defined as a p-value < 0.05.

## Results

Forty-one patients (mean age 63 ± 13 years, 31 male) were enrolled and their final diagnoses were HCC (n = 18), cholangiocarcinoma (CCC; n = 6), metastatic tumor (n = 10), neuroendocrine malignancy (n = 1), and benign lesion (n = 6). The primary sites for hepatic metastasis were colorectal (n = 5), biliary (n = 4), and stomach (n = 1). The benign lesions comprised abscess (n = 1), chronic hepatitis (n = 1), focal steatosis (n = 1), hemangioma (n = 1), steatohepatitis (n = 1), and unknown (n = 1, spontaneous disappearing during follow- up).

**Fig 2:**
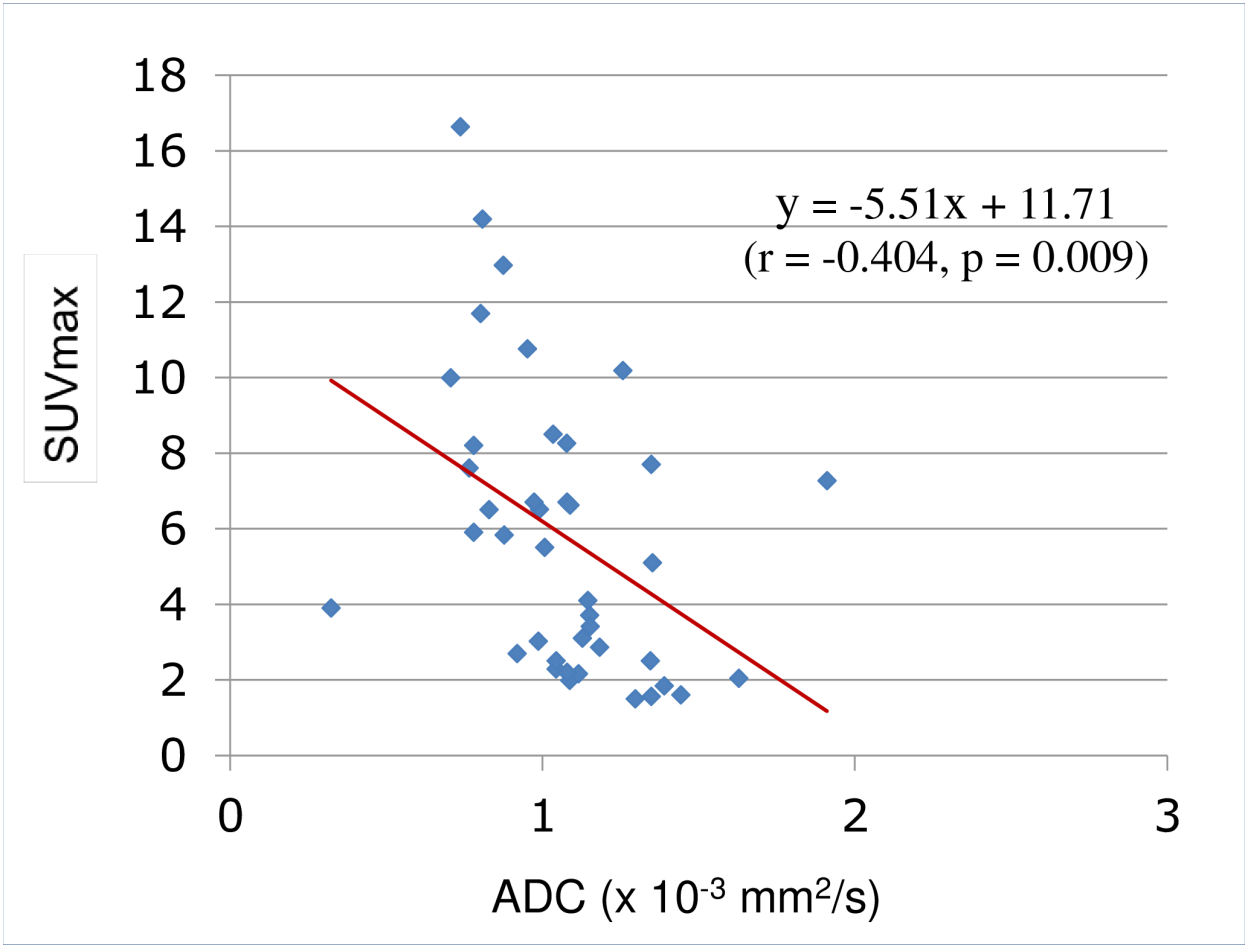
Inverse correlation between SUVmax and ADC. Correlation analysis with scatter plots graph show moderate but significant inverse correlations between SUVmax and ADC (p = 0.009). SUVmax, maximum standard uptake value; ADC, apparent diffusion coefficient.

**Fig 3:**
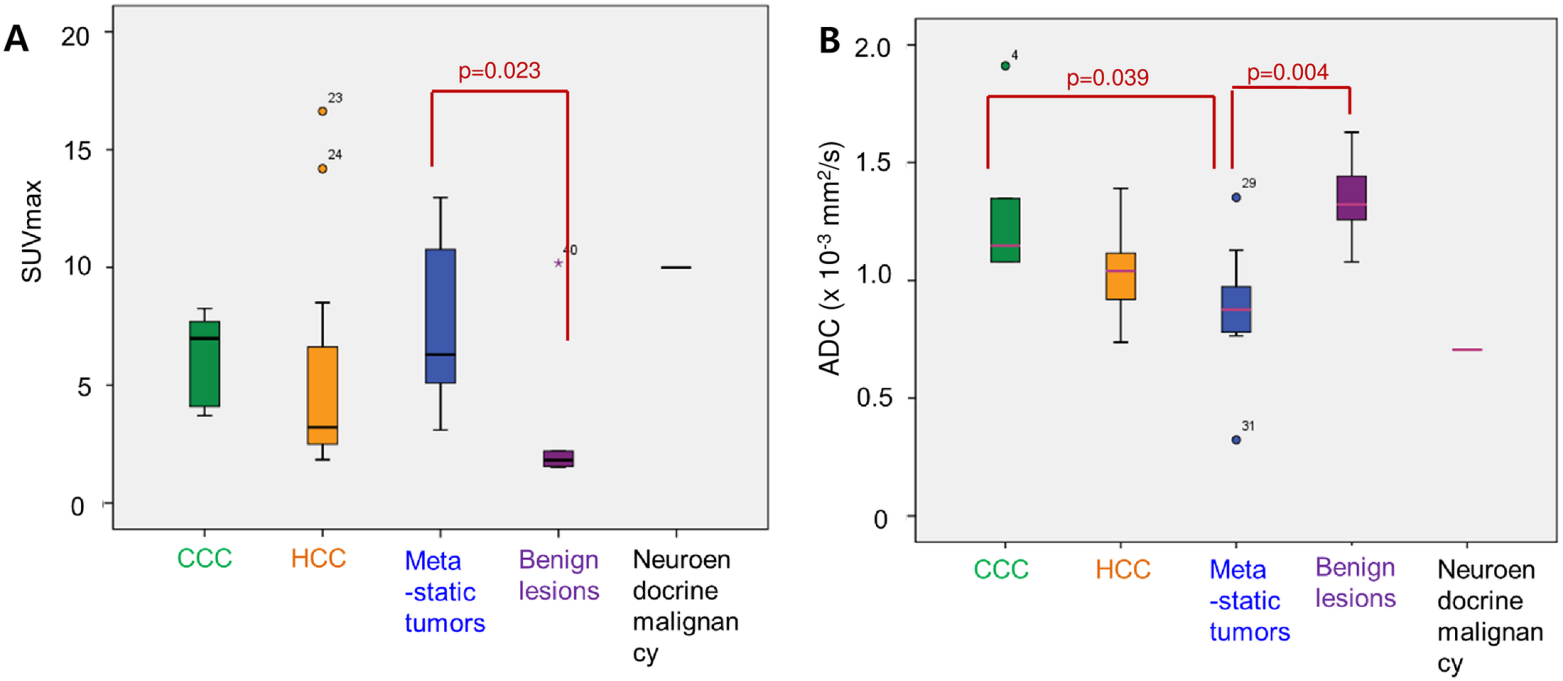
Box plots of SUVmax and ADC values of 41 hepatic tumors. (A) Distribution of SUVmax in various types of hepatic tumors. SUVmax is higher in metastatic tumors than in benign lesions (p = 0.023). (B) Distribution of ADC in various types of hepatic tumors. Metastatic tumor shows lower ADC than benign lesions (p = 0.004) and CCC (p = 0.039). Each box plot shows median (thick lines), quartiles (upper and lower box boundaries), and extreme values (whiskers) within a category. ADC, apparent diffusion coefficient; CCC, cholangiocarcinoma; HCC, hepatocellular carcinoma.

The mean SUVmax for all the tumors was 5.81 ± 3.79 and the mean ADC was 1.069 ± 0.278 × 10^−3^ mm/s^2^. The SUVmax was higher for malignancy (6.26 ± 3.71) than for benign lesions (1.82 (1.5,10.18); p = 0.008) and the mean ADC was lower for malignancy (1.022 ± 0.266 × 10^−3^ mm/s^2^) than for benign lesions (1.323 (1.079, 1.629) × 10^−3^ mm/s^2^; p = 0.003; Table 2). Overall, SUVmax showed a negative correlation with ADC (r = -0.404, p = 0.009; Fig 2). SUVmax by subtype was 3.22 (1.84, 16.63) for HCC, 6.99 (3.71, 8.26) for CCC, and 6.30 (3.10, 12.97) for metastatic tumors. ADC was 1.039 (0.737, 1.390) × 10^−3^ mm/s^2^ in HCC, 1.148 (1.078, 1.911) × 10^−3^ mm/s^2^ in CCC, and 0.876 (0.323, 1.352) × 10^−3^ mm/s^2^ in metastatic tumors. The SUVmax was negatively correlated with ADC in HCC (r = -0.707; p = 0.001) according to Pearson correlation and metastatic tumors (ρ = -0.612; p = 0.012) according to Spearman’s correlation analysis. SUVmax was higher in metastatic tumors than in benign lesions (p = 0.023). The ADC was lower in metastatic tumors than in CCC (p = 0.039) and benign lesions (p = 0.004); ADC was also lower in HCC than in benign lesions, but not significantly (p = 0.06; Table 3, Fig 3).

**Table 2:** Quantitative parameters of malignant hepatic tumors and benign lesions.

	Malignant tumor	Benign lesion	p-value*
**number**	35 (male 28)	6 (male 3)	
**age (years old)**	65 ± 11	51 ± 16	0.056
**SUVmax**	6.26 ± 3.71	3.18 ± 3.44	0.008
**ADC (**× **10**^**−3**^ **mm**^**2**^**/s)**	1.022 ± 0.266	1.342 ± 0.185	0.003

Values are mean ± standard deviation, or n

* p-values were calculated using the Mann-Whitney test.

**Table 3:** Quantitative parameters of hepatic tumor subtypes.

	HCC	CCC	Metastatic tumor	Benign lesion	p-value*
**number**	18	6	10	6	
**age (years old)**	60 ± 8	76 ± 10	69 ± 11	51 ± 16	0.012
**SUVmax**	3.22 (1.84, 16.63)	6.99 (3.71, 8.26)	6.30 (3.10, 12.97)	1.82 (1.5, 10.18)	0.018
**ADC (× 10**^**−3**^ **mm**^**2**^**/s)**	1.039 (0.737, 1.390)	1.148 (1.078, 1.911)	0.876 (0.323, 1.352)	1.323 (1.079, 1.629)	0.002

Values are mean ± standard deviation for continuous parametric variables, median and range (min, max) for continuous nonparametric data, or n

HCC, hepatocellular carcinoma; CCC, cholangiocarcinoma; ADC, apparent diffusion coefficient

* p-values were calculated using the Kruskal-Wallis tests.

## Discussion

With the advent of integrated PET/MRI scanners, it is now possible to obtain multiple functional parameters simultaneously, and this may make it easier to characterize complicated tumor biology [13,14]. In this study, we found a negative correlation between SUVmax and ADC in hepatic tumors when assessed by simultaneous PET/MRI examination. Although SUVmax and ADC represent two different properties in cell biology, our results revealed a relationship between glucose metabolism and water diffusivity. Circulating FDG is transported across the cell membrane by glucose transporters (GLUTs) and accumulates in metabolically active cells. SUV is correlated with tumor cellularity as well as differentiation and histologic grade, and can help to characterize malignancy [15–17]. Previous studies have demonstrated that glucose consumption is correlated with cellularity in non-small cell lung cancer and astrocytoma [15,16]. On the other hand, hypercellular areas could impede water diffusion, leading to lower ADC. A number of malignancies have an ADC lower than that in surrounding healthy, inflammatory or scar tissue [8,9]. Other studies have shown that tumor metabolism is correlated with cell proliferation and tumor cellularity [15,16]. Tumors with a high proliferative index have higher cellularity and therefore more restricted diffusion, leading to lower ADC. Therefore, SUV could have a negative correlation with ADC from a proliferation and cellularity perspective.

The relationship between SUV and ADC has been demonstrated in several other type of tumor. Gu et al. [18] reported a negative correlation between SUV and ADC in 33 patients with rectal cancer (r = -0.450), as did Regier et al. [19] in 41patients with non- small cell lung cancer (r = -0.46) and Schwenzer et al. [20] in 41patients with peritoneal carcinomatosis (r = -0.58). More recently, Brandmaier et al.[13] reported a negative correlation between SUV and ADC in 31 patients with primary or recurrent cervical cancer (r = -0.532) using an integrated PET/MRI scan. Consistent with the above findings, our study identified a significant negative correlation between SUVmax and ADC in 41 hepatic tumors, regardless of their histologic type.

Present literature regarding this correlation for hepatic tumors, in particular for non- HCC lesions, is limited. Ahn et al. [21] reported that SUV did not correlate with ADC in 21 advanced HCCs, as did Boussouar et al. [22] in 28 patients with HCC awaiting transplantation. In contrast, our results did show a negative correlation between SUVmax and ADC for both the entire group of hepatic tumors and for the HCC subgroup. We considered several potential factors explaining these differences. First, all three studies included small numbers of patients; thus the characteristics of an HCC in an individual patient were likely to affect the overall results obtained. Further, SUVmax is defined as the highest single pixel value within the entire tumor volume and only considers the most aggressive tumor components, whereas ADC calculates the average pixel value from each pixel in the entire HCC mass in a single slice; this selected slice is taken as representative of the whole tumor. While increased tumor necrosis observed in highly aggressive HCC is responsible for reduced cellularity and increased ADC, SUVmax is unaffected because SUVmax is only measured from the highest uptake within the mass [23]. Since fibrosis in the interstitial tissue of the liver also could affect diffusivity, the different patient populations enrolled in each study could affect results. Second, different measurement methods were used in these studies. Analyzed data sets were acquired simultaneously with our integrated PET/MR scanner, whereas in the previous research PET/CT and MRI were performed separately with a time interval of days in between scans. Further, differences in the magnetic field strengths (Tesla) of the MRI scanner used and in the ADC calculation methods could affect the ADC value obtained.

Another question in the present study was whether the quantitative parameters of FDG PET/MRI can be used to characterize hepatic tumor subtypes. Although there was a significant difference between malignancy and benign disease, subtype analysis did not show any differences that could be helpful in differential diagnosis. Metastatic tumors showed a lower ADC than CCC, and HCC also tended to have a lower ADC than benign lesions. However, there was considerable overlap in SUVmax and ADC between different types of tumors; thus, these parameters would have limited value for differential diagnosis of hepatic tumors. There are few reports in the literature supporting the use of SUVmax or ADC for differential diagnosis of hepatic tumors. A combination of SUVmax and ADC may have better differential diagnostic ability that either parameter used alone; however, the sample size in our study was too small to determine if this is a possibility.

In many types of cancer cells, uptake of FDG depends largely on the expression of GLUT-1 and hexokinase type II [24]. Previous reports have demonstrated that GLUT-1 is highly expressed in CCC but rarely in HCC [25]. In contrast, expression of hexokinase type II is increased in HCC but not in CCC [26]. Zimmerman et al. [27] reported on the range for GLUT-1 overexpression in metastatic hepatic tumors according to their primary origin. Although SUVmax has limitations in determining the hepatic tumor subtypes, it does provide information on the molecular mechanism, such as glucose uptake, rate of GLUTs, and hexokinase activity, involved in the glycolytic pathway. The diffusion of water molecules in cancer is limited by hypercellularity, enlarged nuclei, hyperchromatism, high nuclear-to-cytoplasmic ratio, and reduced extracellular space. These histopathologic characteristics resulted in a decrease in the ADC. However, these histologic features are common in all types of cancers and therefore cannot be indicators of specific subtypes of hepatic tumors. A previous study mentioned that HCC tend to relatively high cellularity with a granular cytoplasm [28]; this may be related to the lower ADC than CCC. We investigated this assumption but could not find clear correlation between HCC/CCC and ADC. Further study with a larger cohort is needed to determine this relationship. The present study represents a single step in a long journey for differential diagnosis by tumor characterization.

Our study differed from previous work in that our SUVmax and ADC data were obtained simultaneously using an integrated PET/MRI scanner; thus biological changes and misregistration artifacts were minimized. Therefore, we are able to confirm the true biological correlation between SUVmax and ADC without temporal or spatial bias. Our study has several shortcomings, including a small patient cohort, a single center retrospective design, and inclusion of various tumor types. Furthermore, we compared SUVmax values from three-dimensional tumor data with ADC values from two-dimensional information; therefore, information in the DWI might be missing that could not be matched with that obtainable using PET. ADC measurements were only performed on the largest cross-sectional diameter of the tumor. This approach was chosen to achieve reliable ADC measurements; however, the largest lesion is not always representative of all the lesions. Finally, we did not analyze in detail the associations between functional imaging parameters and pathologic examination, and we could not investigate the usefulness of these parameters in clinical situations, such as when decision-making is required.

## Conclusion

Our preliminary study validates the proposed negative correlation between increased metabolic activity and water diffusion in hepatic tumors. Simultaneous acquisition of SUVmax and ADC provides complementary information for characterization of hepatic tumors. Further studies are required to examine the correlation between SUVmax and ADC with regard to the underlying histopathology. Future studies may shed new light on these issues and reveal associations between these functional imaging biomarkers in more detail.

## Supporting information

S1 Table Patients characteristics, SUVmax, and ADC values. (DOCX)
