# Supplementary material for: Quantitative assessment of simultaneous F-18 FDG PET/MRI in patients with various types of hepatic tumors: Correlation between glucose metabolism and apparent diffusion coefficient
Source: PLoS One. 2017 Jul 3;12(7):e0180184. doi: 10.1371/journal.pone.0180184 (PMC5495334; doi:10.1371/journal.pone.0180184)
Supplement: S1 Table — (DOCX) [file pone.0180184.s001.docx]

| Patient | sex | age | diagnosis | SUVmax | ADC (× 10^-3^) |
| --- | --- | --- | --- | --- | --- |
| 1 | F | 85 | Cholangiocarcinoma | 7.7 | 1.348 |
| 2 | F | 55 | Hepatocellular carcinoma | 14.19 | 0.8075 |
| 3 | M | 45 | Hepatocellular carcinoma | 1.84 | 1.39 |
| 4 | M | 48 | Hepatocellular carcinoma | 1.98 | 1.087 |
| 5 | M | 50 | Hepatocellular carcinoma | 16.63 | 0.737 |
| 6 | M | 69 | Metastasis from stomach cancer | 5.83 | 0.877 |
| 7 | M | 64 | Cholangiocarcinoma | 4.1 | 1.145 |
| 8 | M | 60 | Hepatocellular carcinoma | 3.02 | 0.987 |
| 9 | F | 59 | Hepatocellular carcinoma | 2.16 | 1.115 |
| 10 | M | 58 | Hepatocellular carcinoma | 8.2 | 0.78 |
| 11 | F | 34 | Benign lesion: hemangioma | 1.5 | 1.297 |
| 12 | F | 72 | Benign lesion: steatohepatitis | 2.04 | 1.629 |
| 13 | M | 62 | Benign lesion: abscess | 10.18 | 1.257 |
| 14 | M | 58 | Benign lesion: Focal steatosis | 1.6 | 1.443 |
| 15 | M | 68 | Hepatocellular carcinoma | 2.29 | 1.044 |
| 16 | M | 72 | Metastasis from colon cancer | 12.97 | 0.875 |
| 17 | M | 81 | Metastasis from gall bladder cancer | 10.76 | 0.952 |
| 18 | F | 74 | Metastasis from colon cancer | 11.69 | 0.802 |
| 19 | M | 71 | Hepatocellular carcinoma | 3.41 | 1.153 |
| 20 | M | 54 | Hepatocellular carcinoma | 6.62 | 1.088 |
| 21 | M | 57 | Hepatocellular carcinoma | 6.51 | 0.991 |
| 22 | M | 53 | Hepatocellular carcinoma | 2.86 | 1.183 |
| 23 | M | 83 | Cholangiocarcinoma | 3.71 | 1.151 |
| 24 | F | 74 | Cholangiocarcinoma | 7.27 | 1.911 |
| 25 | M | 78 | Cholangiocarcinoma | 8.26 | 1.078 |
| 26 | M | 75 | Hepatocellular carcinoma | 2.5 | 1.044 |
| 27 | M | 63 | Hepatocellular carcinoma | 2.69 | 0.91925 |
| 28 | M | 70 | Metastasis from colon cancer | 5.1 | 1.352 |
| 29 | M | 68 | Hepatocellular carcinoma | 8.5 | 1.034 |
| 30 | M | 48 | Benign lesion: chronic hepatitis | 1.56 | 1.348 |
| 31 | M | 71 | Neuroendocrine malignancy | 9.99 | 0.706 |
| 32 | M | 73 | Metastasis from gall bladder cancer | 7.6 | 0.765 |
| 33 | F | 76 | Hepatocellular carcinoma | 6.5 | 0.829 |
| 34 | M | 52 | Metastasis from pancreatic cancer | 3.9 | 0.323 |
| 35 | F | 70 | Metastasis from rectal cancer | 5.9 | 0.78 |
| 36 | M | 46 | Hepatocellular carcinoma | 5.5 | 1.007 |
| 37 | M | 75 | Hepatocellular carcinoma | 2.5 | 1.345 |
| 38 | M | 58 | Metastasis from ampulla of Vater cacer | 3.1 | 1.128 |
| 39 | F | 33 | Benign lesion: spontaneous disappearing during follow- up | 2.2 | 1.079 |
| 40 | M | 44 | Metastasis from colon cancer | 6.7 | 0.973 |
| 41 | M | 69 | Cholangiocarcinoma | 6.7 | 1.079 |
